# Review of the Problems of Diagnosis of Endopelvic Haemorrhage, Its Intensity, Volume, and Duration, and Treatment Methods of Circulatory Injuries and Surgical Hemostasis after Pelvic Fractures

**DOI:** 10.1155/2019/2514146

**Published:** 2019-02-19

**Authors:** Anatoly Lazarev, Kirill Golokhvast, Ivan Borozda

**Affiliations:** ^1^Priorov Central Science and Research Institute of Traumatology and Orthopaedics, 10, Priorov Street, Moscow, Russia; ^2^Far Eastern Federal University, 8 Sukhanova Street, Vladivostok, Russia; ^3^Amur State Medical Academy, 95 Gorky Street, Blagoveshchensk, Russia

## Abstract

*Objective. *The development of pelvic surgeries is now making new evolutionary jump for us to witness; this would be unimaginable without significant achievements in anesthesiology and new technologies of blood replacement.* Methods. *This overview presents analysis of 41 domestic and 20 foreign references sources that are devoted to the problematics of treating patients with fluid injuries of pelvis accompanied by retroperitoneal haemorrhage.* Results. *The following considers details on determination of endopelvic haemorrhage nature, its intensity, volume, and duration, as well as the treatment methods for circulatory injuries and surgical haemostasis.* Conclusions. *Energy of mechanical influence of traumatizing agent determines the damage levels (system disintegration) for pelvic ring, the bone core, and soft tissue mass. Diagnosis tactics and treatment of patients with disintegrating pelvic damage in the end are determined with volumes and intensity of haemorrhage as well as the character of related damage.

## 1. Introduction

The development of pelvic surgeries which is now making new evolutionary jump for us to witness would be unimaginable without significant achievements in anesthesiology and new technologies of blood replacement.

In the meantime, the choice of treatment tactics for emergency cases of pelvic injuries must be reasoned from the point of reanimation safety for the patient—since in these cases the treatment of traumatic shock and blood loss is the main priorities.

All the reference data that is introduced in this overview is an effort of systemizing diverse and at times contradicting information that was accumulated by generations of doctors who treated patients with pelvic injuries at acute periods of traumatic injuries.

Today the high importance of haemorrhage in pathogenesis of traumatic shock is recognized by almost all scientists who deal with problematics of treating difficult cases of polytraumas [1–24].

Acute haemorrhage and shock prevail in all patients who suffered a polytrauma. It is observed in 80% cases of lethal outcomes as the immediate death cause, especially at the prehospital stage [24].

The severity of traumatic shock in pelvic traumas has immediate correlation with haemorrhage volumes and speed. V. M. Shapovalov and others (2000) note upon the analysis of reference data that traumatic shock develops in 30–58,9% of injured; this said, stable (isolated) injuries represent 20,2–30% and unstable ones - 86,5–100% [25].

Frequency of III level shock in severe pelvic fractures was observed in 75,0-86,5% of the injured; 13,5-20,0% of those patients were in terminal condition [25–27].

Massive endopelvic haemorrhages occur in 37,5% of patients with pelvic fractures. Arterial haemorrhage is associated with unstable pelvic fractures with unstable hemodynamics in 10-20% of patients [28]. In 18,1% of death cases of patients with pelvic fractures was associated with arterial haemorrhages [29].

## 2. Allocating Haemorrhage Points in Accordance with Reference Literature and Personally Acquired Data

Massive haemorrhages in pelvic fractures are mainly caused by the specifics of anatomic vascular supply of pelvic bones and anatomy of vasculature [30]. Venous trunks of periosteum are linked immediately to the sinuses of spongy substance. Therefore, vein has both fixed and mobile sections and is usually damaged at the section borderlines. Pelvic bone and periost veins widely inosculate with vein of nearby muscles and visceral organs. Moreover, arteries supply the bone branch out into two final branches that form *«*lacunes*»*, from where the veins take their origins [31]. Therefore haemorrhages in case of pelvic injuries can be continuous and heavy.

Modern references [25, 32–34] highlight the following points of internal haemorrhages in pelvic injury cases:

1. arterial trunks: (most frequent cases) side and middle arteries of edgebone, upper rectal artery, obturative and ileac arteries;
2. venous: prerectal and paravesical plexuses and ileac veins;
3. vessels of spongy bone, which are located in trabecula and do not deflate and become a reason for continuous and heavy prolonged haemorrhages.

O'Neill, Riina, Sclafani, and others (1996) as a result of analysis of massive volumes of information on angiographic research in patients with unstable pelvic injuries determined that arterial damage in the back half-ring is due to injuries with vertical shift mechanism, whereas in the front one is due to collateral compression; they detected numerous haemorrhage points in 57% of patients with unstable hemodynamics (cit. [25]).

Extraperitoneal haematoma in unstable severe fractures of pelvis is often located in fibers of 3-4 sides from abdominal cavity [15, 35–37] and may involve complications: acute kidney failure, mechanical anuria, intussusception, thrombosis of ileac pelvic veins, compartment-syndrome, osteomyelitis, sepsis, and degenerative changes in feltwork [21, 38–40]. Thus, this requires effective methods of stopping haemorrhage in its acute period, as well as prevention of complications in both immediate and acute periods.

The points of massive haemorrhages in retroperitoneal space and endopelvic fiber are frequently the damaged great endopelvic vessels, presacral and perivesical venous plexuses, and major vessels of the spongy pelvic bone [14, 41–43]. Damage of various vessels occurs in 22,1-30% of cases; thus, damage of arteries and great veins is often diagnosed in lethal cases [41, 44].

Stemlah [21], who analyzed the results of 30 medicolegal autopsies of patients who died because of severe polytrauma with pelvic injuries, notes that injuries of inner ileac artery and vein occurred in 8 cases, outer ileac artery and vein in 3 cases, inner ileac vein in 11 cases, cava in 2 and their branches in all cases.

## 3. Intensity and Duration of Endopelvic Haemorrhage 

Gostev [4] suggests data on the intensity of haemorrhage pelvic polytrauma, stating the numbers 800–1000 ml/h.

In major pelvic injuries, especially of its back part, volumes of haemorrhage can reach 3000 ml with intensity of 1000 ml/h [45].

Shappovalov and others (2000), upon the analysis of reference literature, note that fractures of the wing of heckle bone, branches of pubic bones without shifts of fractured segments, have haemorrhages with volumes from 200 up to 500 ml [25]. If such fractures are diagnosed with shift of fractures segments and ripped pubic symphysis, the volume increases up to 700–1000 ml, in cases with double vertical fractures of Malgen type up to 1500–2500 ml, and in double-side vertical fractures up to 2000–3500 ml.

The research of Burgess and coauthors shows that even in cases of relatively stable injuries with collateral compression (with fractures in the back half-ring) daily haemorrhage volumes were up to 4.760 ml and continued for up to 7 days [25].

As for the vertical unstable pelvic injuries, the work of Pohlemann (1994) shows numbers of total haemorrhage volumes equal to 10.089 ml [29].

Thus, the intensity of haemorrhage depends on the level of damage of spongy pelvic ring (acetabulum, back parts of ileac bone); their fractures form the so-called bleeding bone wound [46]. The more fractures and the bigger the area of this bone wound, the more intense the haemorrhage.

The overview of domestic and foreign literature that was presented by Dyatlov (2001) highlights in detail the modern perception on pathogenesis of endotissual haemorrhage in cases of pelvic fractures [8]. In particular, it was experimentally determined that retroperitoneal space may contain 4 liters of liquids under pressure (according to other authors — 5 liters) with undamaged pelvis, and in case of fractures or destabilization of pelvic ring, open retroperitoneal space and laparotomy – 20 liters.

Experimental research of Kiselev (1950) shows that 3 liters of liquid can be injected during 1 hour through the blood vessel of 1.5 mm and under pressure of 10 Mm Hg. This said hematoma of 1 liter takes the entire pelvis, 2 liter – crawls up to kidneys through the back side of abdominal and to the belly ring at the front side, 3 liter – above the perinephric fiber, and 4 liter – spreads almost up to diaphragm [25].

Experiments on corpses show that diastasis of pubic abarticulation with a size of 10 cm and disruption of rectal-ileac abarticulation by 3 cm increases the pelvic volume by % [8]. This said pelvic volumes in patients who did not have their fractured sections stabilized prior to laparotomy was bigger by 26% [47].

Symptom complex called *«*diagnostics triada*»* was suggested by Dyatlov (2006) [46] for the purposes of early diagnostics of life-threatening massive haemorrhage in retroperitoneal space as a result of rupture of great pelvic vessels.

Triada includes the following:

(1) trauma mechanism — blunt trauma from the side, from the front, back or their combination or vehicle runover in the pelvic area;

(2) typical medial shift of the sharp edge of the distal piece of fractured pelvic bone in above-and supra-acetabular fractures, shift of the pelvis half due to fracture or dislocation;

(3) combination of this data with rapid increase of negative dynamics of laboratory indicators of red blood and ineffectiveness of intensive and full-volume and speed of infusive therapy: with remaining for more than 2 hours critical arterial pressure (70–60/50–40 Mmhg) and drop of hematoglobulin, erythrocyte by 2–4 times and anastalsis of hematocrit by 2–4 during the first 2 hours after the beginning of intensive infusive therapy with mandatory exception of haemorrhage in abdomen, chest, and skull.

Having this symptom complex in cases of injured pelvis ring or acetabulum allows giving diagnosis *«*damage of great pelvic vessels*»*, which requires immediate surgery in retroperitoneal space.

Dyatlov considers pelvic injuries that are accompanied with rapidly growing hematoma of testicles to be another threatening clinical diagnosis, which requires immediate surgery to stop haemorrhage in case of obturator artery damage [46].

According to Shapovalov (2000), stop of bleeding at the spongy bone occurs with full alignment of wounded surfaces and adequate compression in-between, which reaches values of 340-350 N [25].

According to our data [14], alignment of wounded bone surfaces and stable osteosynthesis is already an effective method of surgical hemostasis.

In case of failure of infusive therapy and terminal condition of a patient or III level shock due to haemorrhage with systolic pressure under 60-65 Mm Hg, it is vital to eliminate damage of great pelvic vessels. Only surgery can save the patient, since continuation of such hypotension leads to an irreversible shock [14, 46].

In this situation it is necessary to administer or expand laparotomy in order to revise endopelvic and retroperitoneal hematomas and give treatment (bandaging, clipping arteries, tamponade of retroperitoneal pelvis) [48] and conduct mandatory immediate pelvic ring stabilization, with any effective method, which increases pressure to 60-90 Mmhg according to our data [14].

However, this phenomenon is most likely the result of centralization bloodstream and not decreasing intensity of haemorrhage. Only combination of bandaging of inner ileac arteries with stabilization of pelvic ring allowed us to obtain positive results. Without stabilization of pelvic rings patients died not within first hours at hospital, yet within first three days.

## 4. Volumes of Haemorrhage, Physiological Response to Haemorrhage, Conditions for Stopping Haemorrhage

During the experimental research of dependence of endotissual haemorrhage severity on the character of injuries, we obtained the following results [2].

(1) Locations of bleeding points in pelvic injuries in the ascending order are the following:

- bone,
- bone+vein,
- bone+artery,
- bone+vein+artery.

(2) In cases of simultaneous bone and vessel injuries the intensity of haemorrhage increases from 16,1±0,3 to 25,7±0,2 ml/min—relatively stable ones, and from 16,1±0,3 to 28,6±0,2 ml/min—with unstable fractures. This allows making an oblique conclusion that isolated ruptures of pubic and rectal-ileac articulation can be accompanied with less intense haemorrhage then in the front and back parts of pelvis with spread of pubic bones under 2 cm.

(3) During studying the dependence of haemorrhage volumes and maximal pressure inside pelvis on the type of pelvic ring injury in the conditions of biochemical experiment, the following results were obtained:

- stable damage and ruptures were accompanied with haemorrhage of 670±125 ml with maximal pressure in pelvis equal to 150±8 Mm Hg;
- relatively stable injuries- 890±150 ml (135±11 Mm Hg);
- unstable damage - 1230±180 ml (120±8 Mm Hg).

This evidences lowering blood pressure in a pelvic hematoma and its growth to be proportional to the level of pelvic instability.

The obtained data were correlated with the minimal values of systolic blood pressure in cases of traumatic shock (70 Mm Hg) and normal values of central venous pressure (120 Mm Hg).

Based on the stated above data it can be concluded that pressure in pelvis that is caused by the retroperitoneal hematoma can be an obstacle only for stopping the venous haemorrhage in pelvic area since the levels of central venous pressure do not exceed 120 Mmhg.

At the same time retroperitoneal hematoma cannot be an obstacle for the further development of arterial endotissual haemorrhage, since the pressure in its cavity does not exceed 150 Mmhg and the minimal values of systolic blood pressure are around 70 Mmhg (952 Mmaq).

Simultaneously, retroperitoneal hematoma blocks blood outflow through the lower cava and even serves as *«*venous tourniquet*»* which only increases the blood pressure.

Since haemorrhages from the injured pelvic areas (vascular lacuna) have mixed arteriovenous character, it is obvious that, with injuries of pelvic ring which are accompanied with pelvic bone fractures even without damage to the great arterial vessels, endopelvic haemorrhage has *«*uncontrollable*»* character and without proper treatment it will be lethal.

In such case, common clinical term *«*pelvic volume*»*, which implied some sort of limited space and was introduced by Moss and Bircher in 1996, is only suitable with regard to spread of venous haemorrhage and does not describe precisely the abilities of endopelvic hematomas uncontrollably and fatally developing during arterial haemorrhage.

From this point of view, it is more rational to describe this process as chimney effect [34], when pelvic haemorrhage spreads in a cephalic manner, above m. psoas or along the gluteus as a result of damaged fiber-tissue cases with a risk of development of pelvic or abdominal compartment-syndrome. These cases are often seen as abdominal damage. Since retroperitoneum is not a closed space, the pressure caused by factitious tamponade does not represent clinical value [49].

Quite interesting data that was presented by A. N. Smolyar (2012) is based on the analysis of treating 34 patients with rupture of ventral aorta aneurysm and formation of acute widespread retrocecal hematoma. At the same time the author notes presence of moderate continuous correlation and linear correlation between volumes of retrocecal haemorrhage and intra-abdominal pressure. With maximum volume of retrocecal haemorrhage of 2385 ml, intra-abdominal pressure increases to the I-II level of intra-abdominal hypertension and, therefore, cannot be the only reason for the compartment. Smolyar (2012) considers that only a combination of a few factors, such as massive infusive therapy, development of retroperitoneal haemorrhage, or abdominal cavity surgery, can lead to the intra-abdominal hypertension. Even though the intra-abdominal hypertension syndrome was diagnosed only in 0,5% of patients with closed abdominal injury, lethal rate in this pathological condition is significantly high [50].

Smith, Ziran, and Morgan (2007) [34] present analysis of effectiveness of various methods of stopping haemorrhage in case of disintegrate (relatively stable and unstable) pelvic injuries (Table 1).

The data stated in Table 1 can serve as another argument in favor of active surgical tactics in cases, when the damage artery is the definitely determined point of pelvic haemorrhage.

Such profuse (arterial) haemorrhages represent 10-20% of all injured with pelvic traumas and usually such patients die on a spot or during transportation to the hospital [32].

Data obtained through our research [2] allowed creating the scale for prognosis of injury severity in polytrauma that is convenient for the quick calculation of haemorrhage volumes and severity of traumatic (Table 2).

Upon summary of all the above said, it can be highlighted that endotissual pelvis haemorrhage can be stopped with the following mechanisms: lowering pressure at the moment of traumatic shock, tamponade of veins and vascular lacunas in case of retroperitoneal hematoma formation, and antishock fixation of pelvic bones.

At the same time damage of major arterial trunks leads to the rapidly developing uncontrollable haemorrhage, which can be stopped only through surgery.

## 5. Correctional Methods for Circulatory Injuries

Since endotissual haemorrhage has continuous character prior to stabilization and tamponade of pelvic and retroperitoneal cavities, replacement of blood components has to have advanced character, at least 140% of initially determined haemorrhage volume.

Methodology of calculating volumes of lost blood is most thoroughly developed at the Hannover Medical School. Hemoglobulin concentration is the first parameter to determine upon the arrival of the patient to the hospital. The obtained value is subtracted from the average values for humans, which estimated the amount of lost hemoglobulin. This value is multiplied by the average amount of blood in the human body and the result is divided by the normal hemoglobulin levels. This is the way to obtain volumes of lost blood at the moment of patient's arrival to hospital* V=Hb(n) – Hb(patient)∗V(n)/Hb(n)*. Example: at the moment of male patient's arrival to hospital, hemoglobulin levels are at 90 g/l. Calculation example: V=(150-90)х5.000/150=2.000 ml.

It is determined that by the moment of hospitalization the survived patients lost 2768 ml (from 1046 to 4151 ml) of blood, and the ones who died — 2716 ml (from 1549 to 4016 ml). Then the calculation goes as follows: within the first minutes it is necessary to replace the amount that is not less than the amount of lost blood. Within the first 3 hours the survived patients had 3.7 liters of blood replaced (from 1.5 to 6.6 liters), and the ones who died — 5.5 liters (from 2.1 to 11.7 liters of blood) [29].

Treating massive haemorrhages (with the exception of stopping it), if the urgent and intensive therapy starts already at the prehospital stage or during transportation of the patient, is administered with fluid volemic solutions and crystalloids.

Continuous or started at the admission or reanimation room, transfusion therapy that is adequate in volumes, speed and, content implies administration of 2–3 liters of blood substitutes in a form of colloids of crystalloids that are being injected under pressure with sum speed of 200–300 ml/min. This said 3 liters of blood substitutes are better to be administered within the first 10 minutes. Then at least 2 liters of plasma are injected along with fresh-cell blood with shelf life of less than 2 days. It is considered that this amount of blood products is sufficient for the 2/3 of patients with pelvic trauma and undamaged from the back abdominal infusion. However, in some cases, 5–10 liters of blood products were necessary and with open traumas the amounts are even bigger—from 14.8 to 36 liter over the entire period of treatment [9, 51].

Haemorrhage losses must be replaced with blood products, tempo of replacement must exceed the speed of blood loss, and duration of blood replacement must be equal to duration of haemorrhage. Volume of infusion therapy must exceed haemorrhage volumes depending on its scale: with haemorrhage of 40% of total blood circulation — by 2–2.5 times, 50% of TBC — by 3 times [14].

At the same time, there are warnings on overwhelming the circulation, which is just as dangerous as the hypovolemia and this implies strict count of solution amounts that are being administered into the blood stream and determination of hourly diuresis (at least 100 ml/h without stimulation).

In case of technical opportunity for collecting autologous blood from the cavities, reinfusion is irreplaceable. In cases of extreme exsanguinating, massive reinfusion (over 1 liter) shall be seen as the main reanimation treatment [9, 17].

All by itself infusive replacement therapy cannot and shall not be considered without immediate outer stabilization of pelvic ring and, in accordance with indicators, tamponade of pelvic cavity.

## 6. Methods of Surgical Hemostasis

In case of detection of clinical and/or roentgenologic data on pelvic trauma that influences stability of pelvic ring, antishock pelvic bow or outer fixation device must be applied within the first 30–60 minutes after admission to a hospital [52].

In case of no possibilities to install the above-listed devices, mainly, stabilization of pelvic ring is administered with pelvic bandaging or special pelvic sling, which technology was developed at the Kirov Military Medical Academy [53, 54].

Gilfanov and coauthors [5] suggest data on the use of С-bow Ganz (which is advised in those rare favorable cases when there is an outer-rotational injury of pelvic ring – “clear open book,” and there is no threat of prolapsing fixators into the pelvic cavity through the fracture line) and commonly increased systolic pressure in patients by 30–35 Mmhg.

In other clinical situations it is necessary to use core-type devices for outer fixation of pelvis that allows stable fixation of only front part in most cases [44, 55].

Unfortunately, the majority of structures for outer fixation with front frame, opposite to C-frame Ganz, interfere with laparotomy.

A variety of combinations of the outer frame of the device allow a slight shift of outer frame downwards, which opens new opportunities for laparotomy with stabilized pelvis [53, 56, 57].

In such a way, the thesis that was at some point suggested by Dyatlov is that each laparotomy has to be administered with presence of traumatologist and outer-pelvic stabilization still has urgent topicality [46].

Emergency application of apparatus of external fixation, along with antishock frame Ganz, is proposed by Litvin [16]. The author gives an example of fast stabilization of blood pressure at the levels of 110/60 within 2 hours in patients with severe polytraumas.

In case when administered laparotomy shows damage of abdominal peritoneum, and there is existing connection between hematoma cavity and abdominal cavity, as well as in cases when external fixation and massive infusions do not lead to patient's stabilization within 30 minutes, the only proper choice is tamponade of pelvic cavity. 8-10 towelettes are placed in the back part of borderline in order to fulfill the cavity tightly [42, 58, 59].

Only in cases of angiographically verified ruptures of arterial trunks it can be advised to use active surgical tactics on bandaging the vessel [60]. Most popular criteria for choosing treatment are as follows: haemorrhage from the artery with diameter under 1.0 mm usually stops on its own, from 1.5 to 3 mm (in accordance with some data – up to 5 mm) — endermic embolization of vessel is applied, in case its diameter does not exceed 3 mm the opportunity for open artery ligation is considered [25].

Technique of selective venous embolization requires high level of preparation of angiosurgant—besides necessary equipment and time for surgery—since the consequences of this manipulation can be unpredictable and in a form of vast necrosis of muscles [35, 61].

## 7. Conclusion

Energy of mechanical influence of traumatizing agent determines the damage levels (system disintegration) for pelvic ring, the bone core, and soft tissue mass. Diagnosis tactics and treatment of patients with disintegrating pelvic damage in the end are determined with volumes and intensity of haemorrhage as well as the character of related damage.

Isolated pelvic multifractions are accompanied with endotissual haemorrhages, which in their turn all by themselves (with due stabilization of fractured parts) are not life-threatening in case of no other bleeding points besides pelvic area.

The most widespread haemorrhage point in cases of pelvic ring is in the damaged venous vessels: venous presacral and paravesical plexuses, inner and outer ileac veins and their affluxion, as well as sponge bone lacunes, which are the points of mixed haemorrhage and do not have deflating borders and in case of nonpelvic points of bleeding can be endangering life.

At the prehospital stage, bone stabilization must be administered via bandaging or pelvic sling.

Upon admission patient must be moved only once. Patient is placed on the X-ray clear shield or surgical table in the antishock surgical room.

Among methods for selecting options for surgical stopping of haemorrhages there is immediate external stabilization of pelvic ring with repositioning in order to align edges of bone wounds and limiting pelvic volumes.

In case of lacking effectiveness of intensive transfusion therapy during 30–45 minutes, it is vital to administer laparotomy in order to revise endopelvic and retroperitoneal hematomas and use another treatment (bandaging, artery clipping, and tamponade of retroperitoneal pelvic cavity) that involves mandatory emergency stabilization of pelvic ring via any effective method.

Tamponade of retroperitoneal cavity allows straightening blood pressure in hematoma cavity with pressure in bleeding vessels; moreover, it allows neutralizing haemorrhagic effect that arises during diaphragm movement. On its own tamponade in pelvic cavity without stabilization is inefficient. In the majority of cases without stabilization, C-frame can be administered. In case the damage of back sections of ileac bone does not allow conducting stabilization, external stabilization with C-frame, it has to be conducted via device of external fixation.

Stabilization of pelvic ring must be prior to laparotomy, which in its turn is destabilizing the fractured pieces and provokes further haemorrhage.

It is a mistake and a life endangerment to postpone stabilization of hemodynamics of pelvis with C-frame or with external fixation device prior to patient's condition stabilization.

Only stable pelvis can guarantee stabilization of hemodynamics at acute stage of severe trauma that is moreover accompanied with massive damage of pelvic ring.

## Figures and Tables

**Table 1:** Treatment options for patients with pelvic and hemodynamic instabilities.

Treatment	Advantages	Disadvantages	Effectiveness
Factitious tamponade	no	Effective only in hemodynamically stable patients	yes
		In case of compartment damage	no

MAST	Direct compression (sizing-down the cavity) of pelvic ring and lower limbs	Access limitations to the damaged area Possible complications	no

Internal iliac artery bandaging	No	Full blown collateral	no

Pelvic girdle	Direct compression (sizing-down the cavity) of pelvis without limiting access to the damaged area Biomechanical effectiveness	unknown	possible

Angiography ∖ embolization	No necessity of open access to the retroperitoneum. Isolated haemorrhage can be stopped without surgery.	Arterial source of haemorrhage is discovered only in 10-20% of cases. Time-consuming. Dangerous with development of deep tissue necrosis.	possible

Temporary aorta pressing	Effective in acute situation	Limitations on time	yes

External fixation	Easy and fast administration of stopping haemorrhage by alignment of bone wounds, decreasing pelvic volume Prevention of repeated haemorrhage	Access limitations to the abdominal area. Low-efficiency in C – type damages	yes

Direct stopping of arterial hemorrhage	Stopping hemorrhages from great vessels	Manpower effort	yes

C – frame	Stabilization of back parts – base for tamponade	Special endeixis Possible complications	yes

Internal fixation after exploratory laparatomy.	High biomechanical effectiveness	Special endeixis Manpower effort Experience necessary	yes

Note MAST: medical antishock trousers (pneumocompression).

**Table 2:** Prognosis of traumatic shock severity in pelvic polytrauma.

Injury type	Haemorrhage volume (ml)	Points
*Pelvic fractures*		

Type А	500	10
Type В	1500	30
Type С, acetabulum fracture	2500	50

*Subsequent injuries*		

*Closed fractures*		

Wrist, forearm, foot	750	15
Shoulder	1500	30
Hip	2000	40
Rib	250	5
Brisket	1500	30
Spondyle	2000	40

*Open fractures, wounds*		

Segment fracture	+ 500	10

*Chest*		

Aeropleura	500	10
Overwrought valvular pneumothorax	500×k (k=5)	50
Hemopleura minor	500	10
Hemopleura medium	1500	30
Hemopleura major	2500	50

*Damages of inner organs of abdominal cavity and pelvis*		

Hollow viscus	500	10
Parenchymatous organ	2500	50

Patient age		

18-50 years	×k (k=1)	-
>50 years	×k (k=1,5)	-

*Time since the moment of trauma till the beginning of antishock treatment*		

≤30 min	×k (k=1)	-
30-60 min	×k (k=1,5)	-
≥60 min	×k (k=2)	-

*Prognosis (*traumatic shock severity)		

Favorable (moderate severity)	1000	<20
Questionable (severe)	1.000-2.500	21-50
Unfavorable (extremely severe)	2.500-5.000	51-100

Notes:

(1) Neurosurgical trauma is not includes in the scheme

(2) 1 point = 50 ml of hemorrhage.
